# Simplified pulse wave velocity measurement in children: Is the pOpmètre valid?

**DOI:** 10.1371/journal.pone.0230817

**Published:** 2020-03-27

**Authors:** Saïd Bichali, Alexandra Bruel, Marion Boivin, Gwénaëlle Roussey, Bénédicte Romefort, Jean-Christophe Rozé, Emma Allain-Launay

**Affiliations:** 1 Pediatric Cardiology Unit, Nantes University Hospital, Nantes, France; 2 Pediatric Nephrology Unit, Nantes University Hospital, Nantes, France; 3 Clinical Investigation Center CIC 004, INSERM-Nantes, Nantes University Hospital, Nantes, France; 4 Department of Neonatal Intensive Care, Nantes University Hospital, Nantes, France; San Raffaele Roma Open University, ITALY

## Abstract

In population exposed to cardiovascular risk, aortic stiffness is an important marker which is assessed by carotid-to-femoral pulse wave velocity (PWV). In childhood, the validated applanation tonometer SphygmoCor® can be used to measure PWV, but is limited in routine practice by the child’s cooperation and operator’s experience. An alternative device, the pOpmètre® is validated in adults and rapidly measures finger-to-toe PWV using 2 oxymeter-like sensors. The aim of this study is to validate the pOpmètre® device in children aged between 4 and 8 years. We compared simultaneous PWV measurements of the two devices, SphygmoCor® and pOpmètre®, in a training group, using the Bland-Altman method. Then we proposed an algorithm to correct pOpmètre® PWV (PWVpop). Finally, we validated this new algorithm in a validation group of children using the Bland-Altman method. This prospective study enrolled 26 children in the training group. Mean PWVpop was 3.919 ± 0.587 m/s and mean SphygmoCor® PWV was 4.280 ± 0.383 m/s, with a difference of -0.362(CI95%(-0.546;-0.178)) m/s. A new algorithm was defined using transit time (TTpop): corrected PWVpop (m/s) = 0.150/TTpop(s) + 1.381*Height(m) + 1.148. We enrolled 24 children in the validation group. Mean corrected PWVpop was 4.231 ± 0.189 m/s and mean SphygmoCor® PWV was 4.208 ± 0.296 m/s with a corrected difference of 0.023(CI95%(-0.086;0.131)) m/s. With this algorithm correction, we found an agreement between PWV measured by the SphygmoCor® and the pOpmètre®, with a difference of less than 10%. Using this algorithm, the pOpmètre® could be used in clinical or research practice in young children exposed to cardiovascular risk. (This study was registered as NCT02991703).

## Introduction

Long-term increase in cardiovascular risk has been well established in children with defined pathologies such as diabetes [1], chronic kidney disease (CKD) [2], and in children born preterm or small for gestational age (SGA) [3]. Multiple parameters are used to estimate this risk, among which arterial stiffness is highly indicative [4, 5]. The gold standard to evaluate regional arterial stiffness is carotid-to-femoral PWV measured by validated methods including applanation tonometry with the SphygmoCor® system in adult population [6–8]. Reference values in healthy 7–8 year old children have been published with normal measured SphygmoCor® PWV (PWVsphyg) range between 3.5 m/s and 5.4 m/s [9, 10]. There is no defined pathological threshold in children whereas for adults, above 10 m/s the patient is considered to be at high cardiovascular risk [11]. Moreover, these values are modified by anthropometric factors: age, gender, height [9], blood pressure [12], and ethnic background [13].

In current practice, pulse wave velocity (PWV) is not performed for children at risk of cardiovascular disease because of lack of adapted material. Indeed, the SphygmoCor® is a common device in adult practice, but is limited by its usability, especially in small children who have to remain still, and its requirement of an experienced operator. In children, a more efficient device is necessary. The pOpmètre® instrument, similar to pulse oximeters, utilizes finger and toe pulse wave sensors to measure PWV using photo-plethysmography and can be suitable for use in children [14]. This technique has already been validated to assess digital pulse volume reflecting peripheral pulse pressure [15]. A good correlation with the reference technique has been published and the detection algorithm and signal processing of the pOpmètre® have been improved and validated in adults [16].

The pOpmètre® has not yet been validated in children for PWV measurement. The aim of this prospective single-center study was to demonstrate the value of this device, first in a training group of children aged between 4 and 8. We then compared pOpmètre® and SphygmoCor® PWV measurements in each child and established a new algorithm, and finally validated it in a validation group.

## Materials and methods

### Ethics statement

The study was registered by the French Commission for Data Protection (National Committee for Informatics and Liberties) and was accepted by the Person Protection Committee (the Ethic authority in the West region in France). The project was registered in ClinicalTrials.gov (Identifier: NCT02991703).

### Population

From January to July 2017, we enrolled children aged between 4 and 8, from all pediatric units of the Nantes University Hospital including Outpatient Care, Surgery, General Pediatrics and Pediatric Specialties. In each age range, 12 children were included with a sex ratio of 1:1. Written parental (and child if appropriate) approval was obtained. The child was not included if he or she presented an incompatibility to physiological PWV measurements including pain and agitation making clinical status non-compatible with the measurements, or other situations which could interfere with the peripheral blood flow: compressive bandages on the measured area, peripheral venous catheter or vascular surgery on the right side of the body, vasoconstrictive treatments and temperature less than 36°. Characteristics of the children, including age, weight, height, peripheral oxygen saturation, signs of good peripheral blood perfusion and heart frequency were noted. The child past medical history of prematurity, SGA, CKD, cardiovascular disease (CVD) or cardiovascular risk factor (CVRF: hypertension, diabetes, dyslipidemia, familial past medical history of cardiovascular disease), and the reason of hospitalization or consultation were also collected.

### Number of patients required by group

We decided arbitrarily that the mean difference between the SphygmoCor® and pOpmètre® PWV had to be less than 10% of the PWVsphyg value, which seems acceptable in clinical practice. As the expected PWVsphyg was 4.5 m/s with a standard deviation (SD) of 0.45, the acceptable mean difference was 0.45 m/s between PWVsphyg and pOpmètre® PWV (PWVpop) [9, 13]. This corresponds to the ARTERY society recommendations which consider the accuracy of the tested device as excellent when the mean difference is less than 0.5 m/s [17]. With a power of 90% and an alpha risk of 5%, at least 22 patients were required for each group: the training and validation groups. Taking into account the possible measurement difficulties with the SphygmoCor® device, we decided to include 30 children in each group, totaling in 60 children altogether, with equal number of girls and boys as recommended [17].

### Measurement methods

The SphygmoCor® XCEL device (AtCor Medical, Australia) used both a carotid applanation tonometer and a femoral cuff. Although applanation tonometry is considered as the gold standard method, the cuff-based SphygmoCor® XCEL device has been validated in children [18]. It detected carotid and femoral pressure wave form and calculated the delay, called carotid-to-femoral transit time (TTsphyg), between the beginnings of each wave detected successively. Depending on the distance between carotid and femoral pulses, an algorithm transformed this time difference into PWVsphyg. Distances from carotid pulse to cuff upper side and from femoral pulse to cuff upper side were measured after a cuff was put around the right thigh [4]. Other informations were systolic, diastolic and mean arterial pressure on the right arm. Brachial systolic and diastolic blood pressure measurements were taken with a semi-automatic oscillometric device (Dinamap Procare® General Electric, USA).

The pOpmètre® (Axelife sas, France) uses photoplethysmography to detect the arterial pulse waveform in the distal phalanx of the finger and toe. Particular attention was drawn on sensor positioning so that the distal phalanx was in contact with the photodiode. This device measured the pulse wave arrival time difference between the big toe (heart-to-toe TT) and the index finger (heart-to-finger TT) at each heartbeat, called finger-to-toe TT (TTpop) and transformed it into PWVpop [16].

TT and PWV measurements were performed for each child by 2 experienced operators. The child laid back supine with bare feet and arms, at rest in the consultation or hospitalization room [19]. After the SphygmoCor® cuff was put around the right thigh, measurements were started simultaneously on both devices. The carotid-to-femoral distance was measured using the direct method and was multiplied by 80% as recommended [20]. Two SphygmoCor® PWV values were taken and their mean was calculated. At the same time, at least 2 PWV values were taken with the pOpmètre® system on the left side of the body with a third measurement if the difference between the 2 first values was at 0.5 m/s or above. The mean of the 2 closest values on the left side of the body was calculated. This procedure with the pOpmètre® was repeated on the right side after the cuff was taken off. The operators reassured and distracted the child especially when applying the SphygmoCor® carotid probe. The whole procedure was completed in 20 minutes and no follow-up was planned.

### Statistical analysis

In the training group, we compared PWVpop and PWVsphyg using the Bland-Altman method. To validate the pOpmètre® device, the primary outcome measure was the mean difference (DiffPopSphyg) between the 2 simultaneous PWVpop and PWVshyg. The pOpmètre® was considered valid if the 95% confidence interval (95% CI) of DiffPopSphyg was less than 10% of the normal value (ie between -0.450 and +0.450 m/s). We then created an algorithm to calculate the corrected PWVpop. To define the algorithm, we performed a linear regression with PWVshyg as a dependent variable, 1/TTpop and height as independent variables. Coefficients *a*, *b* and *c* in the formula PWVsphyg = *a*/TTpop + b*Height + *c* were determined. In the validation group, with a Bland-Altman test, we compared PWVsphyg and corrected PWVpop, calculated using the algorithm previously defined. Moreover, we performed a sensitivity analysis to include children with aberrant values to the validation group. To assess repeatability, within-patient coefficients of variation were determined for both devices (100*SD/mean). Statistical analyses were performed using SPSS 22.0. Categorical variables were compared using χ2 test and continuous variables using Student t test. A p value under 0.05 was considered significant. When applicable, data were summarized as mean ± SD.

## Results

### Population characteristics

**Fig 1:**
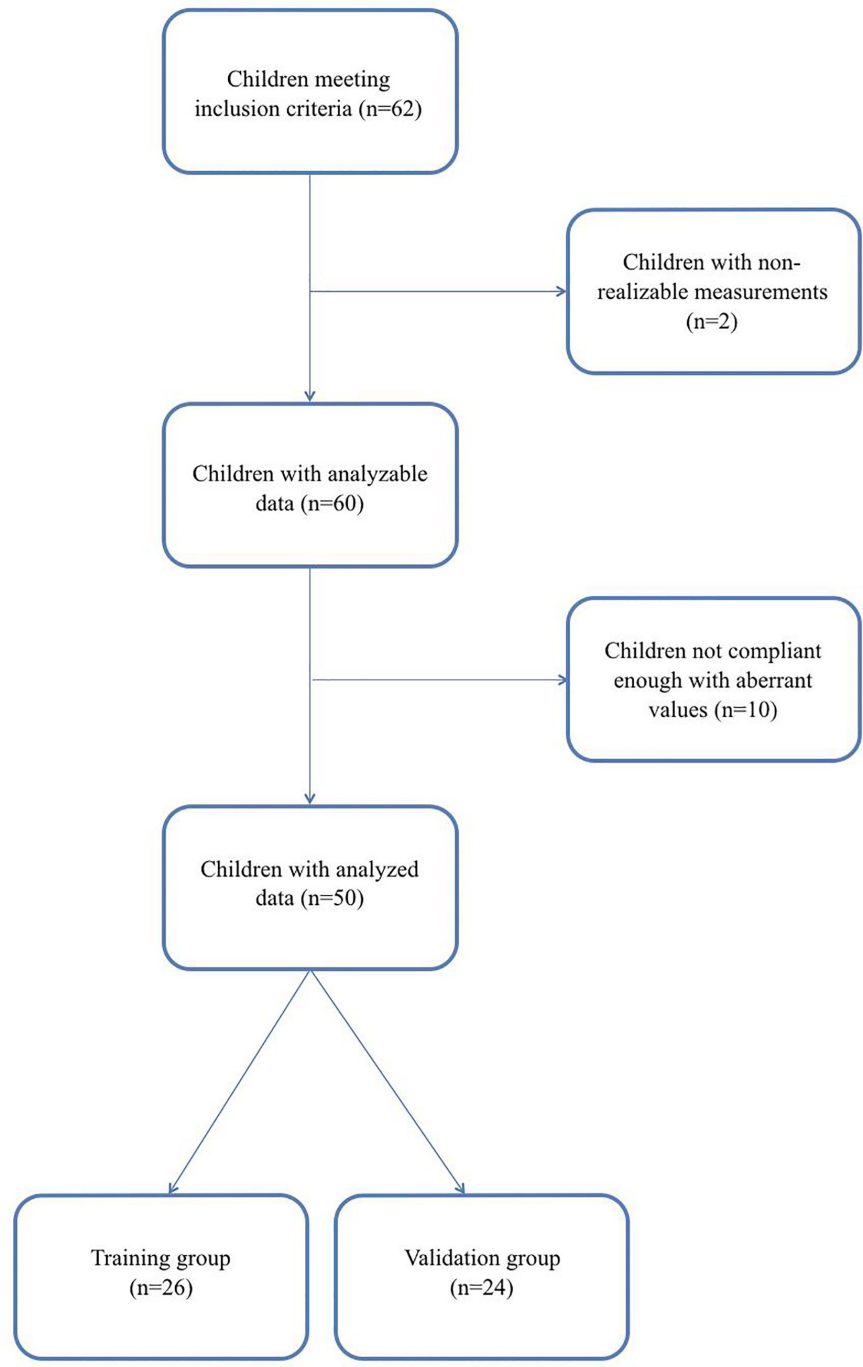
Flow chart.

After exclusion of 2 children due to non-cooperation of the patient, 60 children had analyzable data with 12 children for each year of age between 4 and 8 years old and a sex ratio of 1:1. We then excluded 10 children from analysis, 4 in the training group and 6 in the validation group, due to SphygmoCor® measuring difficulties (Fig 1). Characteristics of the population in both groups are described in Table 1. A large part of the cohort came from pediatric cardiology or nephrology consultation (n = 22, 44%) and 6 children (12%) had a past medical history of SGA or prematurity.

**Table 1:** Children characteristics.

	Training Group	Validation Group	p
**Number of children**	26	24	0.777
**Age (years old)**	6.1 ± 1.5	5.9 ± 1.4	0.620
**Sex ratio (female/male)**	1.2	1.2	0.805
**Weight (kg)**	22.7 ± 5,6	21.8 ± 4,2	0.543
**Height (m)**	1.21 ± 0,10	1.18 ± 0,09	0.258
**BMI (kg/m**^**2**^**)**	15.4 ± 2,0	15.6 ± 1,7	0.611
**Apex-To-Hyoid Bone Distance (cm)**	19.2 ± 1,9	19.6 ± 1.726	0.472
**Hyoid Bone-To-Suprasternal Fossa Distance (cm)**	6.4 ± 1,6	6.0 ± 1.3	0.275
**Suprasternal Fossa-To-Finger Distance (cm)**	62.6 ± 5,3	62.3 ± 6.4	0.904
**Suprasternal Fossa-To-Trochanter Distance (cm)**	39.7 ± 4,2	39.7 ± 5.3	0.963
**Trochanter-To-Heel Distance (cm)**	58.9 ± 6,3	56.9 ± 6,1	0.252
**Heel-To-Toe Distance (cm)**	18.8 ± 1,6	18.0 ± 1,6	0.093
**SpO2 (%)**	98.3 ± 1,6	98.7 ± 1,5	0.487
**Heart rate (beat per minute)**	94.7 ± 13,4	94.0 ± 18,6	0.882
**SBP (mm Hg)**	104.9 ± 11,1	101.8 ± 8,5	0.280
**DBP (mm Hg)**	64.5 ± 11,3	61.4 ± 6,5	0.240
**MBP (mm Hg)**	78.1 ± 10,6	76.1 ± 5,8	0.410
**Number of children with Hypertension**^**a**^	5	1	0.102
**Number of Children with Past Medical History of CKD, CVD, CVRF, SGA or Prematurity**	14	12	0.695

Results are presented as mean ± standard deviation when applicable.

BMI: Body Mass Index, SBP: Systolic Blood Pressure, DBP: Diastolic Blood Pressure, MBP: Mean Blood Pressure, CKD: Chronic Kidney Disease, CVD: Cardio-Vascular Disease, CVRF: Cardio-Vascular Risk Factor, SGA: Small for Gestational Age.

^a^Hypertension is defined as above the 97.5 percentile for height.

### PWVpop versus PWVsphyg in the training group

**Fig 2:**
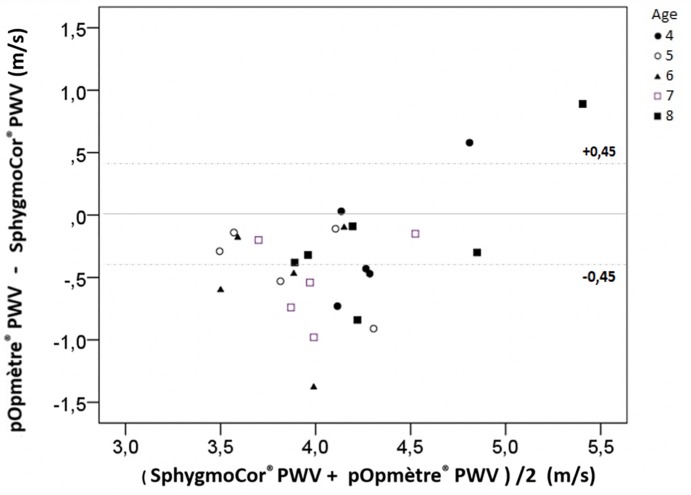
Bland-Altman graph for the 26 children from the training group. Mean ± 2 standard deviations = -0.362 ± 0.958 m/s. PWV: pulse wave velocity.

Among the 26 children of the training group, mean PWVsphyg was 4.280 ± 0.383 m/s and mean PWVpop was 3.919 ± 0.587 m/s (data are available in S1 Table). The 95% CI of DiffPopSphyg was -0.546 to -0.178, inferior to the acceptable limit previously defined. Thus, PWVpop was not validated in the training group, PWVsphyg being underestimated (Fig 2).

### Corrected PWVpop in the training group

The coefficients given by linear regression were *a* = 0.150, *b* = 1.381 and *c* = 1.148. The equation giving corrected PWVpop depending on TTpop and height with the best agreement between PWVsphyg and corrected PWVpop values was then as followed:

$$\begin{array}{l} CorrectedPWVpop(m/s)=0.150/TTpop(s)+1.381\;*\;Height(m)+1.148 \end{array}$$

Mean corrected PWVpop was 4.280 ± 0.214 m/s. The 95% CI of DiffPopSphyg was -0.115 to 0.145, inside the -0.450 to +0.450 interval.

### Corrected PWVpop versus PWVsphyg in the validation group

**Fig 3:**
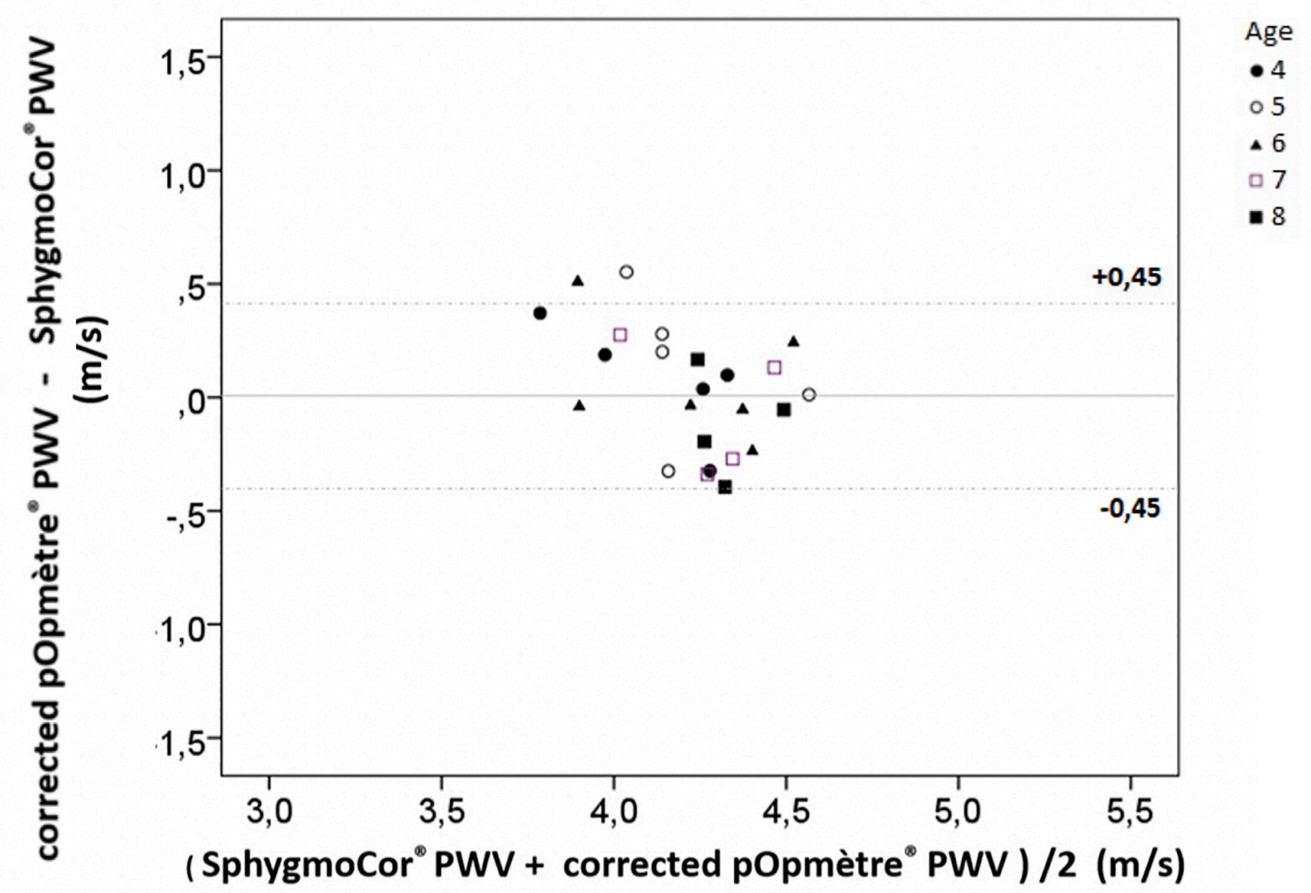
Bland-Altman graph for the 24 children from the validation group. Mean ± 2 standard deviations = 0.023 ± 0.540 m/s. PWV: pulse wave velocity.

We tested the corrected PWVpop in the validation group (data before correction are available in S2 Table). Among the 24 children, mean PWVsphyg was 4.208 ± 0.296 m/s and mean corrected PWVpop was 4.231 ± 0.189 m/s. The 95% CI of DiffPopSphyg was -0.086 to 0.131, inside the -0.450 to +0.450 interval. PWVsphyg and corrected PWVpop showed a good agreement (Fig 3). Including the children with aberrant values to the validation group (n = 30), the 95% CI of DiffPopSphyg was -0.186 to 0.135, inside the -0.450 to +0.450 interval. Corrected PWVpop and PWVsphyg for the 50 children of the 2 groups are presented in Table 2.

**Table 2:** Fifty-child corrected PWVpop and PWVsphyg depending on age and gender.

Age and gender (n)	CPWVpop (m/s)	PWVsphyg (m/s)
**4 years old (10)**	4.273 ± 0.215	4.256 ± 0.310
**5 years old (10)**	4.220 ± 0.165	4.096 ± 0.373
**6 years old (11)**	4.196 ± 0.205	4.145 ± 0.348
**7 years old (9)**	4.231 ± 0.158	4.284 ± 0.277
**8 years old (10)**	4.365 ± 0.243	4.460 ± 0.332
**All children (50)**	**4.256 ± 0.202**	**4.246 ± 0.342**
**All years, male (23)**	**4.272 ± 0.267**	**4.195 ± 0.318**
**All years, female (27)**	**4.243 ± 0.126**	**4.289 ± 0.362**

Results are presented as mean ± standard deviation.

CPWVpop: corrected pOpmètre® Pulse Wave Velocity, PWVsphyg: SphygmoCor® Pulse Wave Velocity.

### Repeatability

Within-patient coefficients of variation for repeated measures were 3.1%, 3.2%, and 2.9% for TTpop, TTsphyg and PWVsphyg respectively. Of note, left and right corrected PWVpop showed a very good agreement among the 43 children with available data: mean difference between left and right PWVpop was 0.018 ± 0.214 m/s.

## Discussion

We did not find a good agreement before correction between the pOpmètre® and the SphygmoCor®. However, after correction by a new proposed algorithm, we obtained a good agreement between the 2 devices in children aged between 4 and 8 years old, in a validation group. Thus, it seems that the pOpmètre®, easy to use in clinic, can be used in this child population.

Detecting signs of arterial stiffness as soon as possible is an issue for pediatric specialists. An appropriate, validated and reproducible method to detecting arterial stiffness in children is highly desirable. Validation of PWV data in children is ethically not feasible with invasive methods; values in healthy children have been published with applanation tonometry using the SphygmoCor® or the PulsePen® and oscillometry using the Vicorder® or the Arteriograph® [21, 22]. These devices have provided comparable PWV results and have been accepted as reference methods in children down to 6 years old [23, 24]. Values in younger children have been published but have not been validated with a method considered as a reference [21, 25]. Moreover, some data were published with formulas extrapolated in young children aged 4 years old with no actual measurement [10, 26].

The pOpmètre® is a photo-plethysmography method validated in adults [14]; here we validated it in comparison with a reference method specifically in young children (4 to 8 years old). Data issued from the training group were initially disappointing. It could be suggested that this poor agreement was influenced by the longer part of the peripheral arterial tree included in the pOpmètre® measure whereas the SphygmoCor® is limited to carotid-to-femoral distance. However in adults, despite this difference, the correlation between TTpop and TTsphyg was excellent using the actual pOpmètre® optimized signal processing that was used in our study [16]. Although the 2 techniques employed different methods, they both aimed at calculating PWV with a good agreement using a new formula.

In adult, studies validating the pOpmètre®, correlation was better with transit time (TT) than PWV [14, 16]. Moreover for children, formulas extrapolating PWV are often based on height [9, 26]. Hence our proposition of algorithm is simple including only TT and height which is available in a common pediatric consultation. Once this correction was applied, good agreement was obtained in the validation group. Furthermore, the pOpmètre® has shown a high right-left reproducibility which increases its usability in daily clinical practice.

The difficulty in measuring TTshyg and PWVsphyg led to the exclusion of 19% of values in this study. In a study by Keehn et al., data could not be acquired for 22% of children [25]. Indeed, the SphygmoCor® system requires a trained operator and training has shown to improve measurement success [27, 28]. Children should stay motionless during the procedure which is stressful and hard for the youngest, thus signal remains often suboptimal. In our experience, one whole measurement took at least 10 minutes with 2 experienced investigators. In a pilot study including 3-to-5-year-old children, 2 nurses were needed to reassure the child with a long procedure of 15 to 30 minutes [29]. This observation limited our use of the SphygomoCor® in pediatrics hence inviting us to find a more reliable and reproducible method. The pOpmètre® was found to be rapid, feasible, acceptable-by-patient and almost operator-independent [14]. It can be utilized by nurses, who commonly use saturation sensors in children. The only well-known limit is coldness of hands and feet causing vasoconstriction necessitating repeated measure, which can be avoided in a warm laboratory or by rubbing cold extremities [15, 30].

PWV data were presented here by age and gender: these PWV data could not be defined as reference values because of the small size and the heterogeneity of the cohort. Each patient was its own control to compare both methods. Despite the past medical history of some children, we could note that our population had morphological parameters (height, weight, body mass index) correlated to their age because their measurements were compared to the World health organization growth charts and were close to the mean values [31].

The necessity and the impact of evaluating aortic stiffness in children younger than 6 years old could be controversial. However, some PWV abnormalities have been described in groups potentially having an increased cardiovascular risk, as in young children with congenital heart diseases and in infants and neonates whose mother have diabetes or hypertension [32–34]. These PWV values were considered abnormal without defined reference values; we therefore think that validating the pOpmètre® in large cohorts of very young children could allow routine PWV measurement. Indeed, during consultation with the specialist pediatrician, a long-term screening and follow-up could be realized in perinatology networks, pediatric nephrology, and pediatric cardiology for an optimal cardiovascular prevention in children [35–39].

To conclude, the pOpmètre® device has been successfully compared to the reference SphygmoCor® in children between 4 and 8 years old using a new algorithm which includes height and TT. This technique seems to be more appropriate than other devices actually validated for routine follow-up. To allow a reliable use of the pOpmètre®, our data need to be validated in larger cohorts of healthy and ill children as results from adults cannot be extrapolated to children [40].

## Supporting information

S1 Checklist TREND statement checklist. (DOCX)

S1 Table Pulse wave velocities in the training group. (PDF)

S2 Table Pulse wave velocities in the validation group. (PDF)
