# Supplementary material for: Simplified pulse wave velocity measurement in children: Is the pOpmètre valid?
Source: PLoS One. 2020 Mar 27;15(3):e0230817. doi: 10.1371/journal.pone.0230817 (PMC7100956; doi:10.1371/journal.pone.0230817)
Supplement: S1 Checklist — (DOCX) [file pone.0230817.s001.docx]

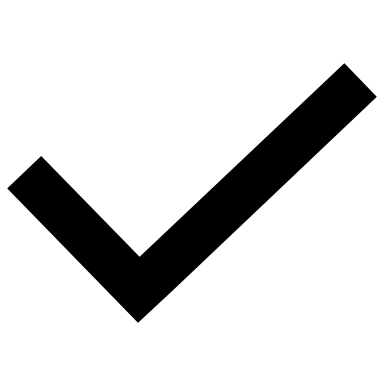

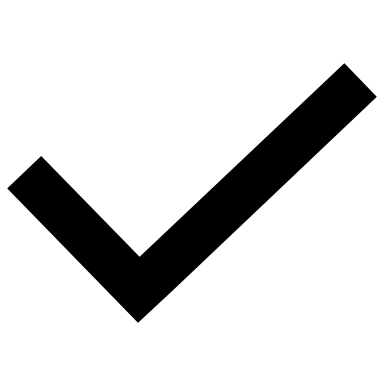

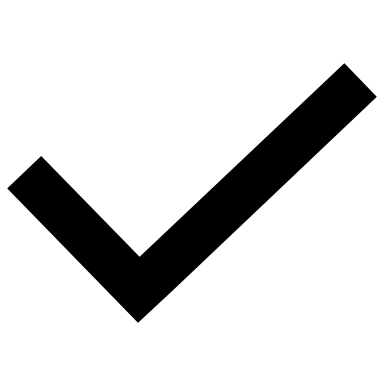

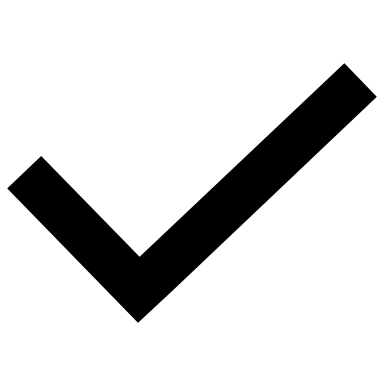

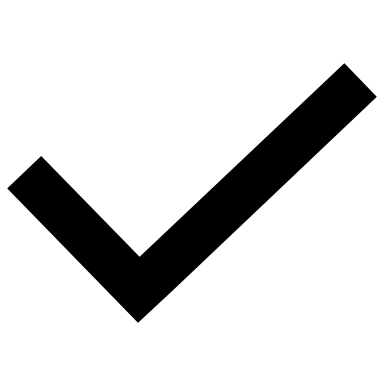

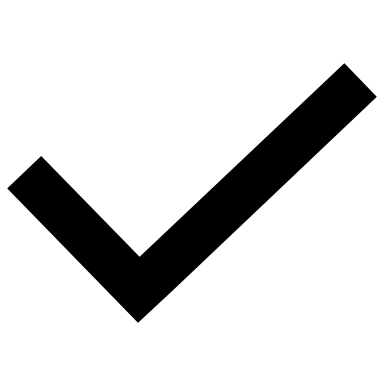

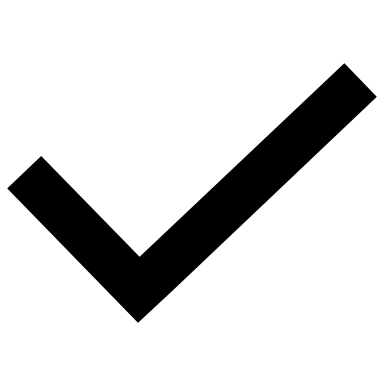

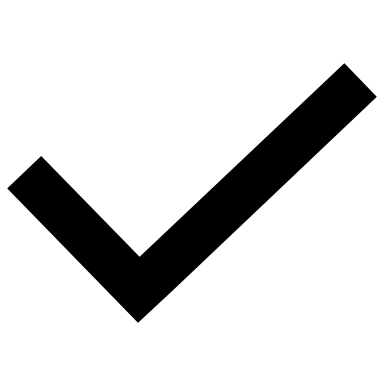

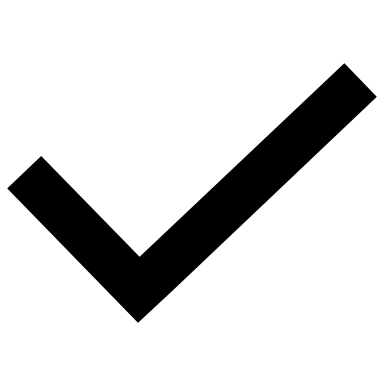

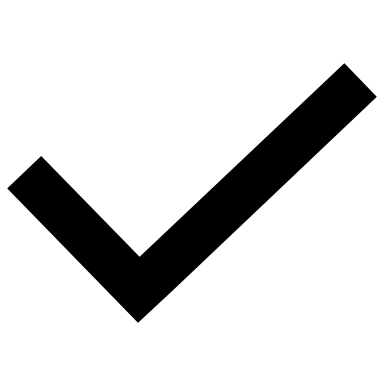

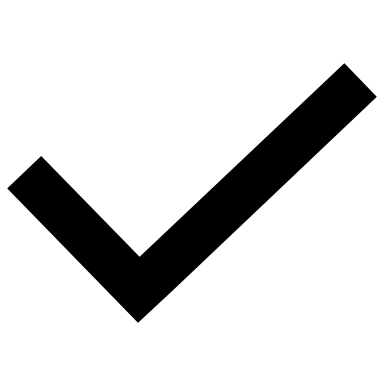

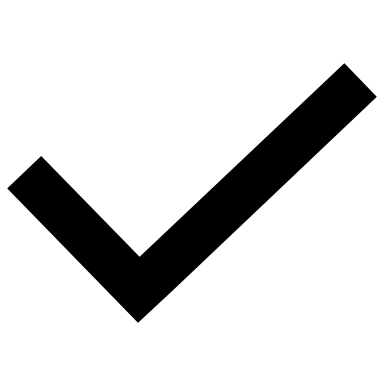

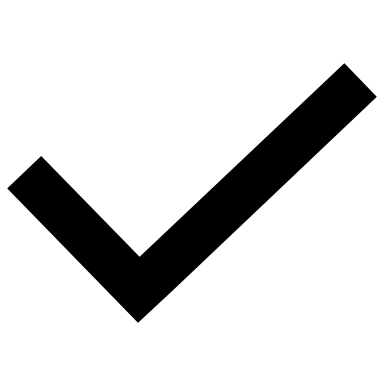

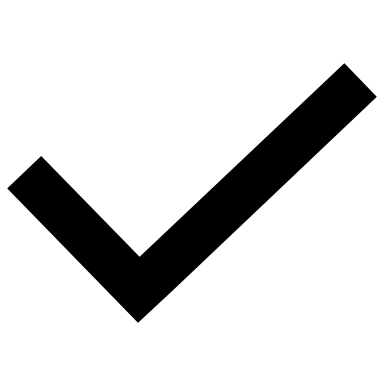

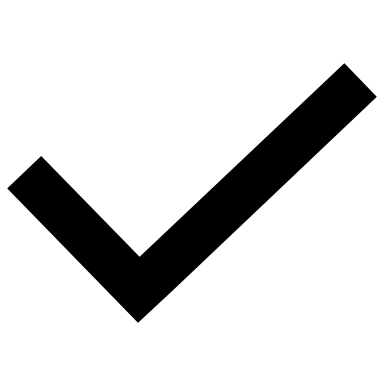

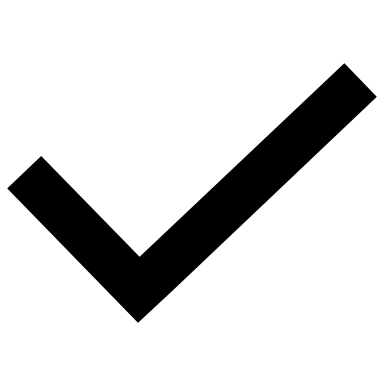

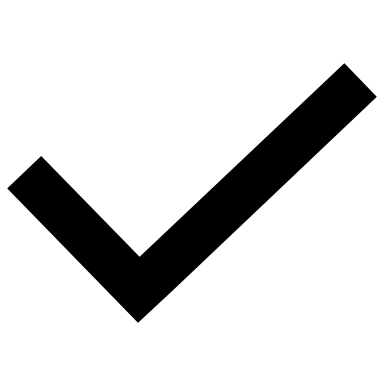

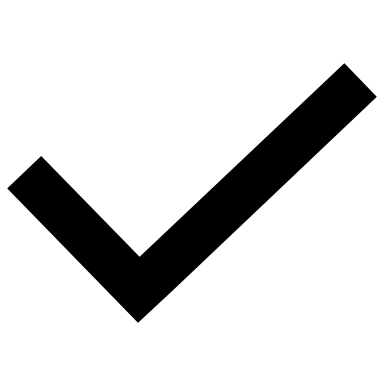

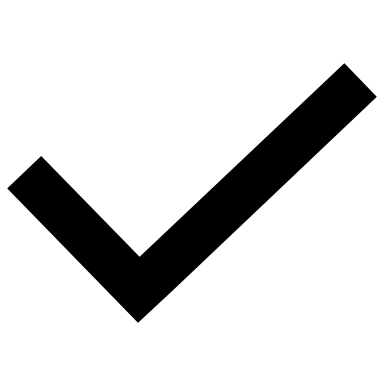

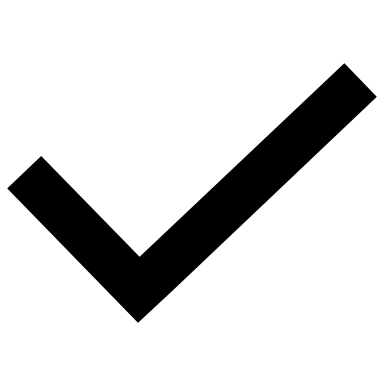

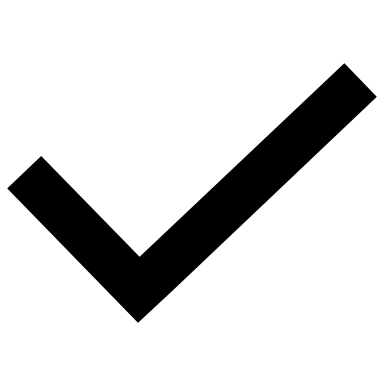

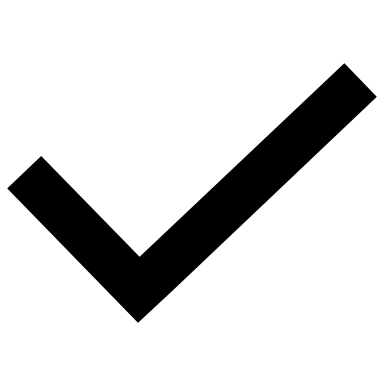

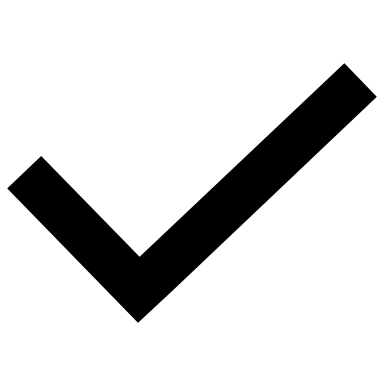

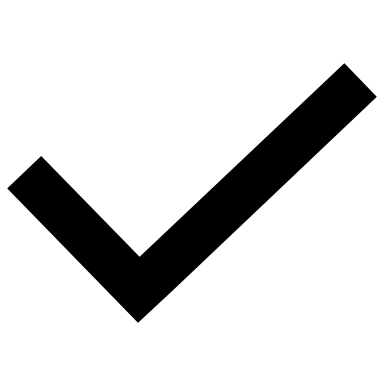

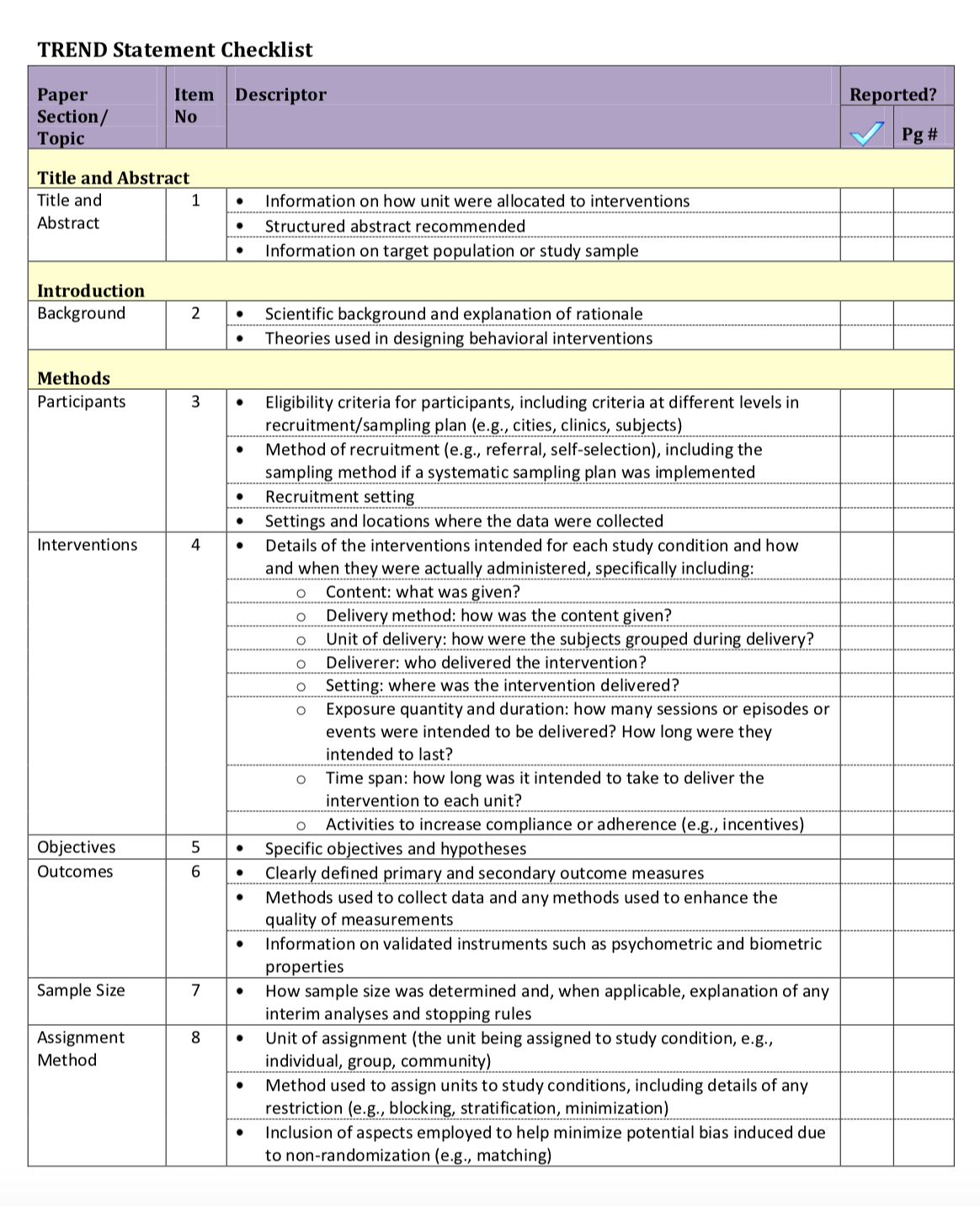


2

2

2

3

4

4

4

4

5

5

5

5

5

5

5

5,6

2

6

5

5

4

5

5


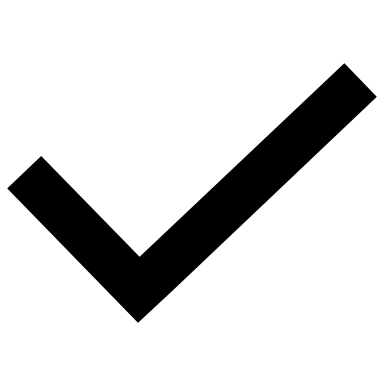

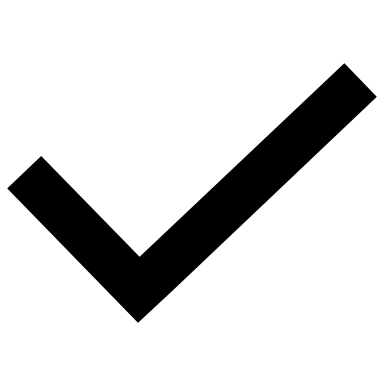

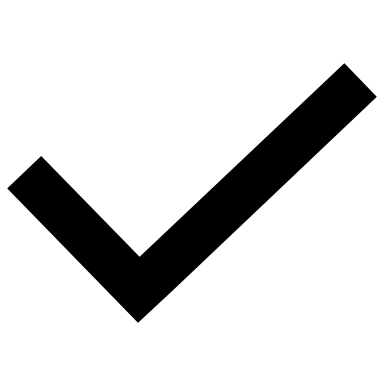

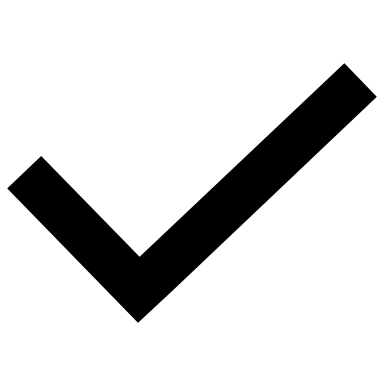

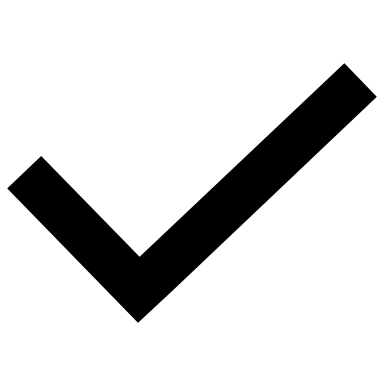

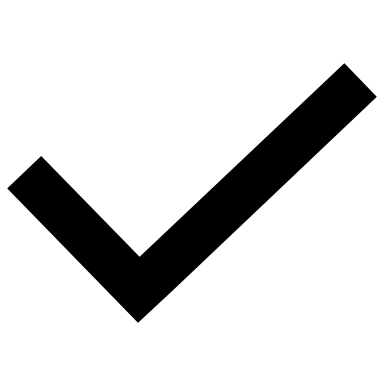

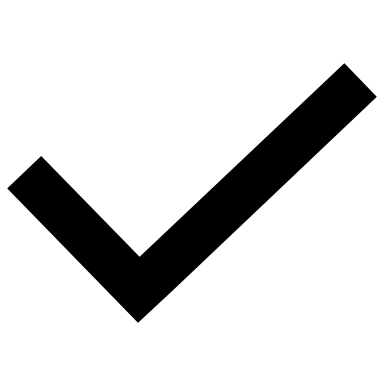

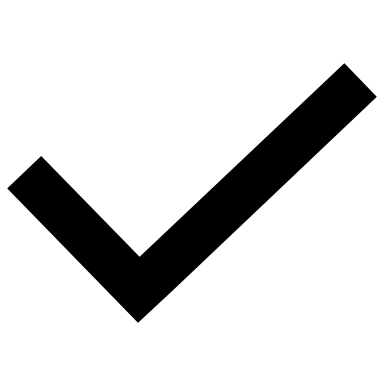

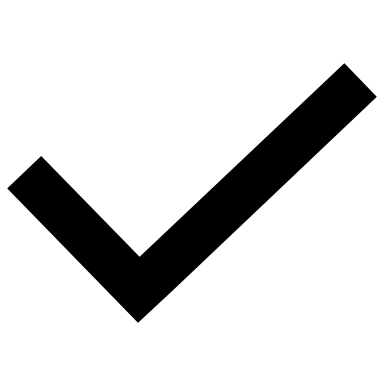

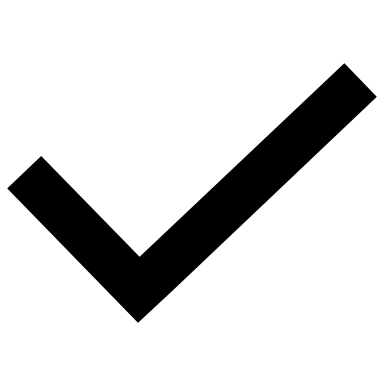

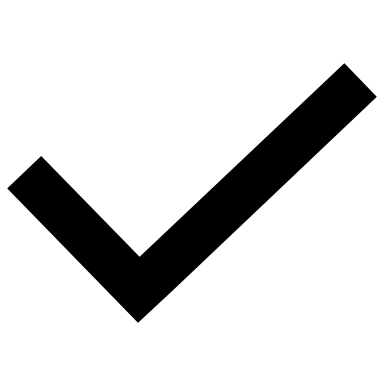

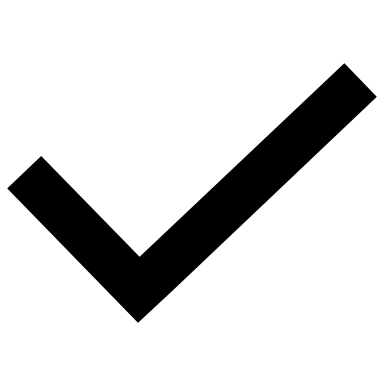

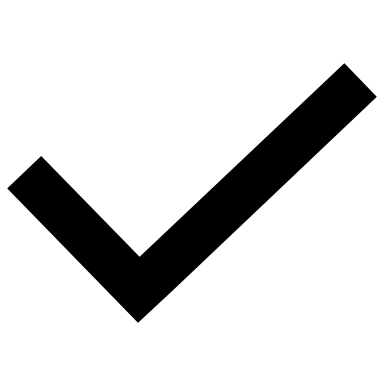

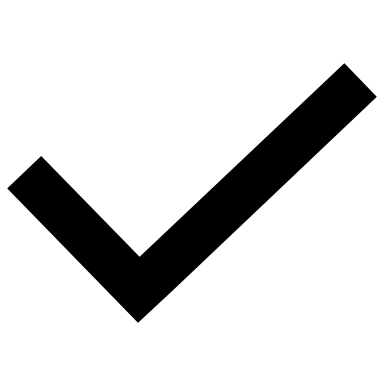

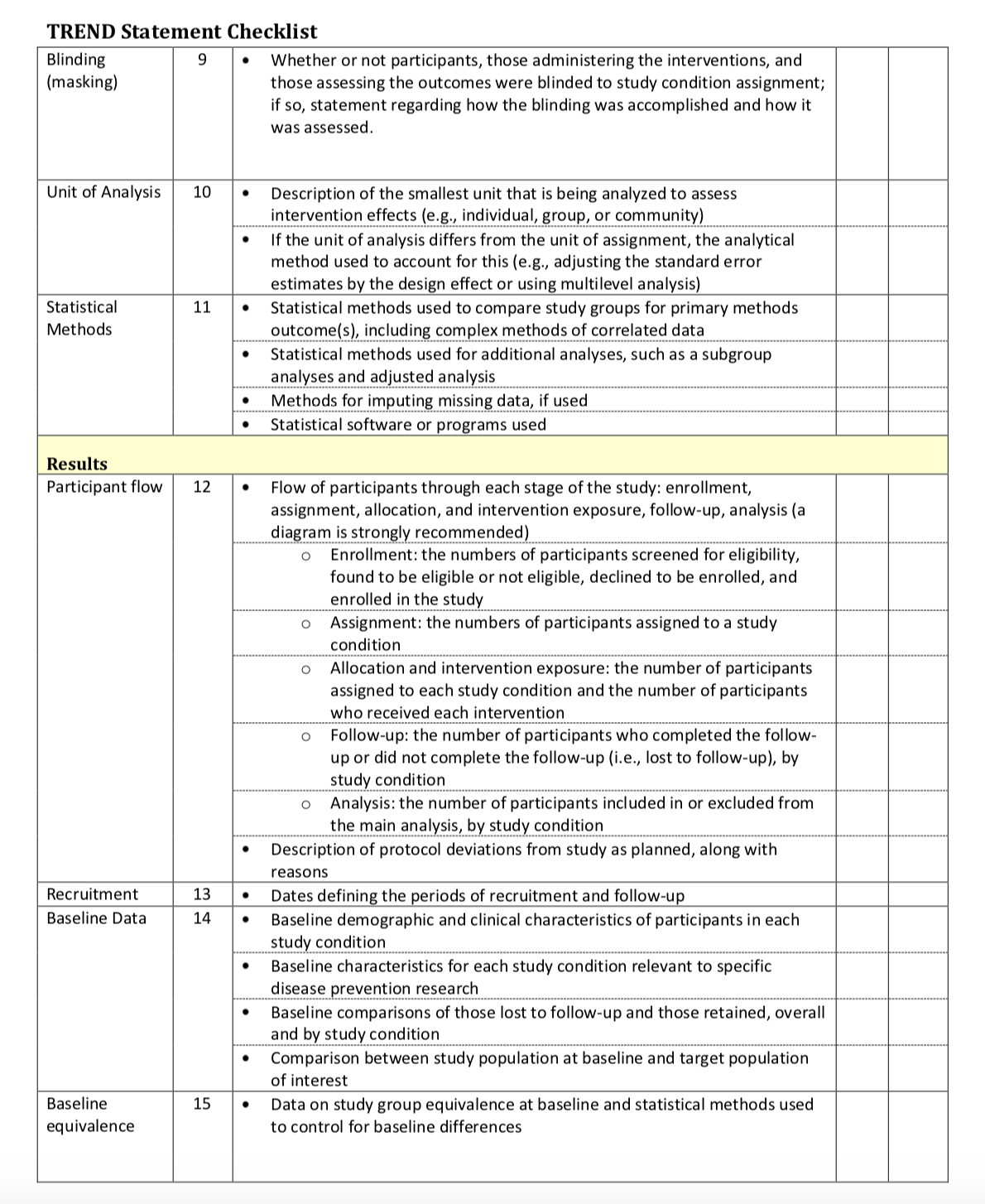


5

5

6

6

7

7

7

7

7

4

7

7

7

7


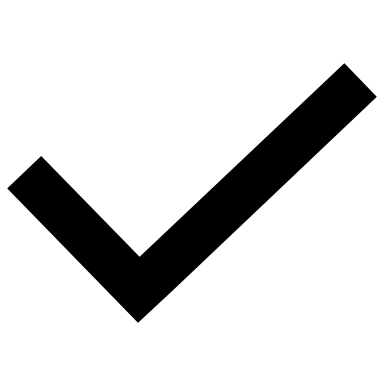

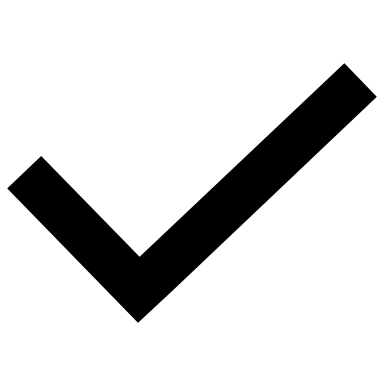

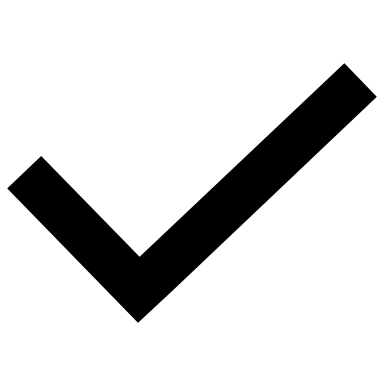

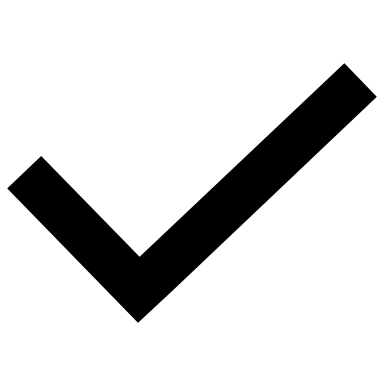

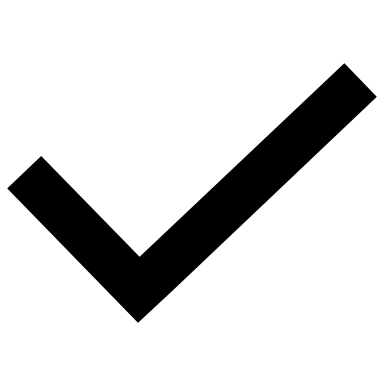

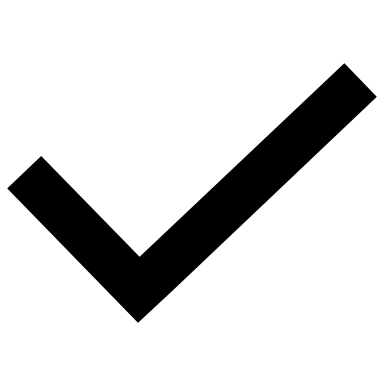

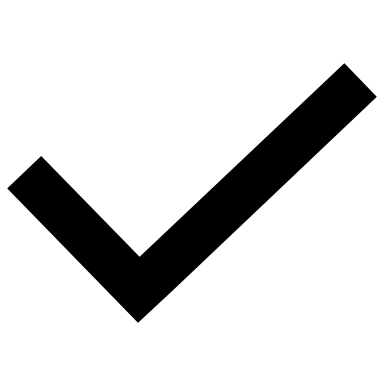

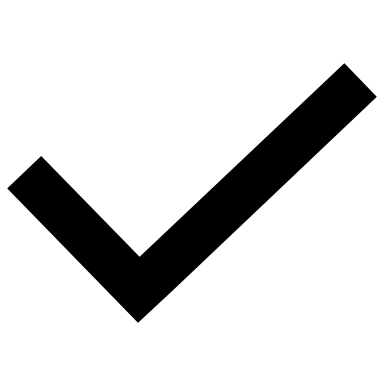

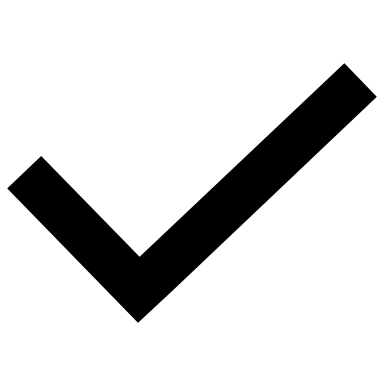

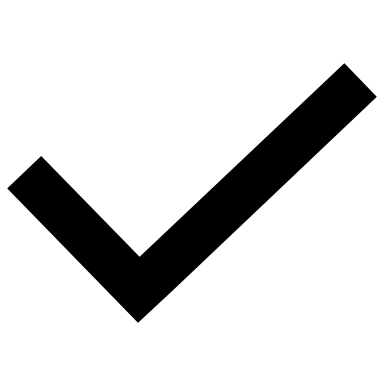

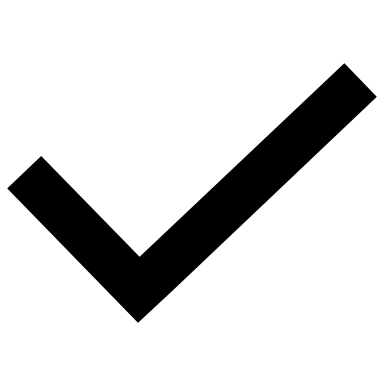

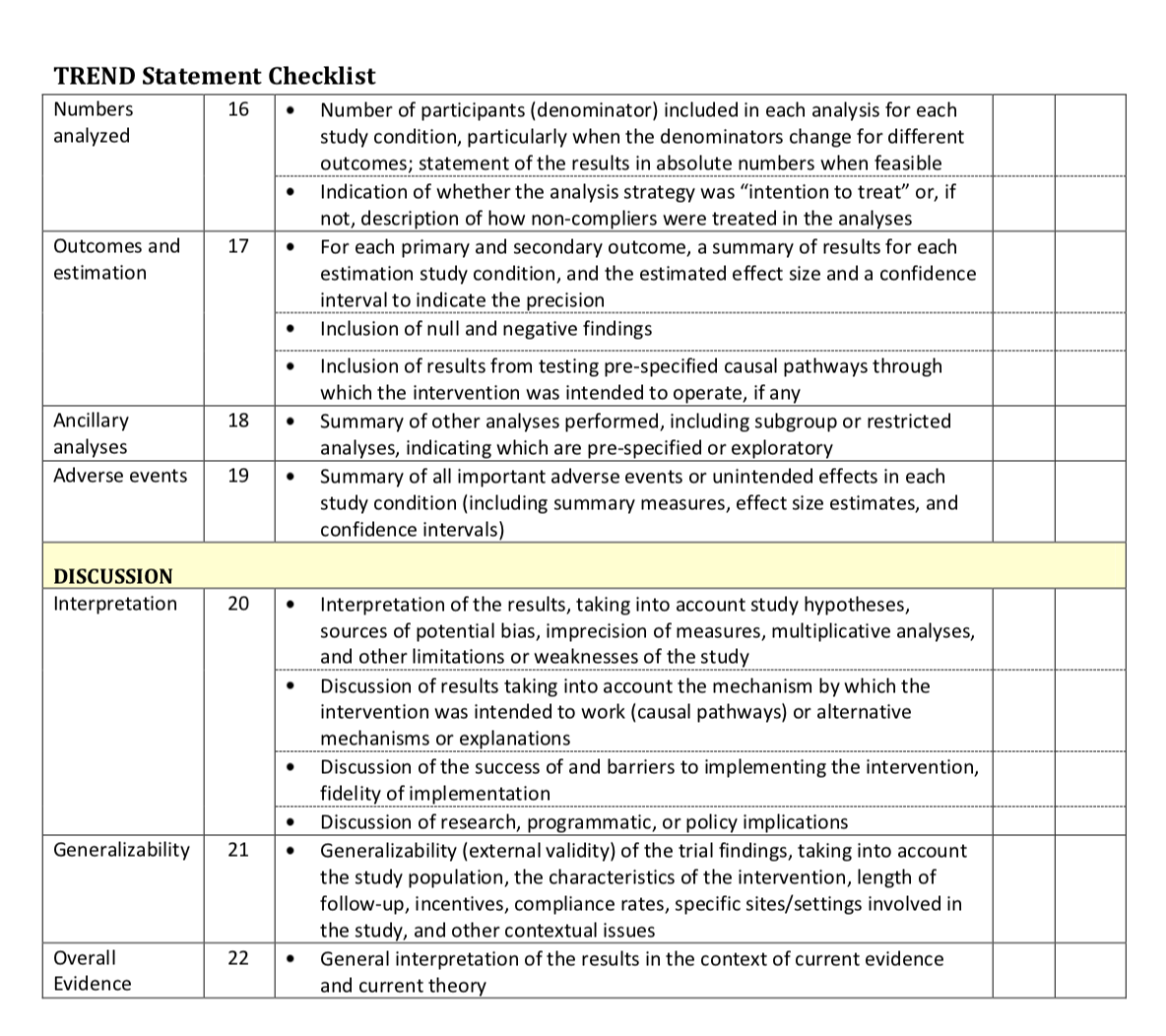


7,8,9

7

7,8,9

8

8

11

10,11

10,11,12

11,12

12

12

From: Des Jarlais, D. C., Lyles, C., Crepaz, N., & the Trend Group (2004). Improving the reporting quality of nonrandomized evaluations of behavioral and public health interventions: The TREND statement. American Journal of Public Health, 94, 361-366. For more information, visit: http://www.cdc.gov/trendstatement/
