# Supplementary material for: Simplified pulse wave velocity measurement in children: Is the pOpmètre valid?
Source: PLoS One. 2020 Mar 27;15(3):e0230817. doi: 10.1371/journal.pone.0230817 (PMC7100956; doi:10.1371/journal.pone.0230817)
Supplement: S1 Table — (PDF) [file pone.0230817.s002.pdf]

S1 Table. Pulse wave velocities in the training group.

Patient Number	Age (years old)	PWVpop (m/s)	PWVsphyg (m/s)
1	6	4.100	4.200
2	4	5.100	4.520
3	6	3.500	3.680
4	8	4.150	4.240
5	4	4.150	4.120
6	5	3.550	4.080
7	8	5.850	4.960
8	4	4.050	4.480
9	4	3.750	4.480
10	6	3.650	4.120
11	8	3.700	4.080
12	8	4.700	5.000
13	5	4.050	4.160
14	7	3.500	4.240
15	7	3.600	3.800
16	8	3.800	4.120
17	4	4.050	4.520
18	5	3.850	4.760
19	6	3.300	4.680
20	8	3.800	4.640
21	6	3.200	3.800
22	5	3.350	3.640
23	5	3.500	3.640
24	7	3.500	4.480
25	7	4.450	4.600
26	7	3.700	4.240

PWVpop: pOpmètre® Pulse Wave Velocity. PWVsphyg: SphygmoCor® Pulse Wave Velocity.
