# Supplementary material for: Simplified pulse wave velocity measurement in children: Is the pOpmètre valid?
Source: PLoS One. 2020 Mar 27;15(3):e0230817. doi: 10.1371/journal.pone.0230817 (PMC7100956; doi:10.1371/journal.pone.0230817)
Supplement: S2 Table — (PDF) [file pone.0230817.s003.pdf]

S2 Table. Pulse wave velocities in the validation group.

Patient Number	Age (years old)	PWVpop (m/s)	PWVsphyg (m/s)
1	4	3.400	3.880
2	4	3.100	3.600
3	8	3.600	4.360
4	6	3.850	4.520
5	8	3.950	4.160
6	6	4.850	4.400
7	5	3.900	4.000
8	7	3.600	3.880
9	6	3.600	3.640
10	5	3.250	4.320
11	6	3.650	4.240
12	6	4.050	4.400
13	8	3.600	4.520
14	4	4.100	4.280
15	4	3.550	4.440
16	8	4.350	4.520
17	4	3.900	4.240
18	5	4.000	3.760
19	6	2.850	3.920
20	7	3.500	4.440
21	7	3.650	4.480
22	7	5.000	4.400
23	5	4.650	4.560
24	5	3.800	4.040

PWVpop: pOpmètre® Pulse Wave Velocity, PWVsphyg: SphygmoCor® Pulse Wave Velocity.
